# "Driving the devil away": qualitative insights into miraculous cures for AIDS in a rural Tanzanian ward

**DOI:** 10.1186/1471-2458-10-427

**Published:** 2010-07-20

**Authors:** Maria Roura, Ray Nsigaye, Benjamin Nhandi, Joyce Wamoyi, Joanna Busza, Mark Urassa, Jim Todd, Basia Zaba

**Affiliations:** 1 Centre for Population Studies, Department of Epidemiology and Population Health, 49-51 Bedford Square, London School of Hygiene and Tropical Medicine, London, WC 1B 3DP, UK; 2 The TAZAMA Project, NIMR, The Tanzanian National Institute for Medical Research, PO Box 1462, Isamilo, Mwanza, Tanzania

## Abstract

**Background:**

The role of religious beliefs in the prevention of HIV and attitudes towards the infected has received considerable attention. However, little research has been conducted on Faith Leaders' (FLs) perceptions of antiretroviral therapy (ART) in the developing world. This study investigated FLs' attitudes towards different HIV treatment options (traditional, medical and spiritual) available in a rural Tanzanian ward.

**Methods:**

Qualitative interviews were conducted with 25 FLs purposively selected to account for all the denominations present in the area. Data was organised into themes using the software package NVIVO-7. The field work guidelines were tailored as new topics emerged and additional codes progressively added to the coding frame.

**Results:**

Traditional healers (THs) and FLs were often reported as antagonists but duality prevailed and many FLs simultaneously believed in traditional healing. Inter-denomination mobility was high and guided by pragmatism.

Praying for the sick was a common practice and over one third of respondents said that prayer could cure HIV. Being HIV-positive was often seen as "a punishment from God" and a consequence of sin. As sinning could result from "the work of Satan", forgiveness was possible, and a "reconciliation with God" deemed as essential for a favourable remission of the disease. Several FLs believed that "evil spirits" inflicted through witchcraft could cause the disease and claimed that they could cast "demons" away.

While prayers could potentially cure HIV "completely", ART use was generally not discouraged because God had "only a part to play". The perceived potential superiority of spiritual options could however lead some users to interrupt treatment.

**Conclusions:**

The roll-out of ART is taking place in a context in which the new drugs are competing with a diversity of existing options. As long as the complementarities of prayers and ART are not clearly and explicitly stated by FLs, spiritual options may be interpreted as a superior alternative and contribute to hampering adherence to ART. In contexts where ambivalent attitudes towards the new drugs prevail, enhancing FLs understanding of ART's strengths and pitfalls is an essential step to engage them as active partners in ART scale-up programs.

## Background

Religious organizations are a fundamental part of the social structure in rural Africa and since the beginning of the HIV epidemic have played a major role providing material and spiritual support to persons living with HIV/AIDS (PLHA)[1,2].

The influence of religion on HIV risk behaviours [3-7] and on stigmatization of PLHA [1,8-11] has received considerable attention. Relatively few studies, however, have explored the links between religious beliefs and attitudes to antiretroviral therapy (ART).

In Tanzania, a quantitative study conducted among parishioners from both rural and urban settings showed that in spite of widespread belief in the ability of prayer to cure HIV, most respondents would *hypothetically *be willing to initiate ART if diagnosed with the infection [1]. In contrast, an earlier study conducted in Uganda showed that 1.2% of *actual *ART users-all of whom where members of Pentecostal churches - had discontinued treatment because they thought that they had been spiritually healed [12]. In a study conducted in Ethiopia *"holy water" *was mentioned as a reason for interrupting treatment as frequently as *"not being able to afford transportation costs" *[13]. While inconclusive on the role of religious beliefs on treatment adherence, a study conducted in Mali found an association between the belief that HIV is a *"punishment from God" *and fatalistic attitudes towards the disease [14].

The rapid growth of Pentecostal-type revivalist churches in Sub-Saharan Africa (SSA) during the 1990 s has coincided with the burgeoning HIV epidemic. An ethnographic study conducted in urban Tanzania described how neo-Pentecostal churches became increasingly attractive in the context of AIDS partly due to the spiritual healing offered in times when biomedical treatment was unavailable [15]. In Nigeria, a detailed ethnography of a revivalist church provided vivid examples of how individuals may turn to religion as a way of coping with HIV [16]. In a context where AIDS symptoms are often attributed to witchcraft [17-20], the presumed ability to combat sorcery has also been reported as a crucial factor contributing to the success of the African Pentecostalism movement [21].

The use of prayer as a means to exorcise spirits and treat HIV has been associated primarily with revivalist churches [1,15,16], although spiritual treatments for HIV are also offered by branches of mainstream denominations, as illustrated in a recent study that describes a *"holy water service" *to combat *"demonic infestations"*. Such services, marketed as *'the Lourdes of Tanzania"*, are conducted in Dar es Salaam and other major cities despite the disapproval of ecclesiastic authorities [22]. The use of traditional medicine to treat HIV symptoms has also been extensively documented in Tanzania and other SSA countries [19,20,23-30].

In our study setting, previous qualitative findings pointed to a need for in-depth investigation of FLs perceptions of HIV treatments [25,30,31]. Within the sample of 17 ART users that we have followed since 2006, the only person who died was successfully responding to treatment but decided to interrupt it after a "laying-on-of-hands" session, which he believed miraculously cured him. In our interviews with village leaders, we heard on several occasions that there were FLs in the ward offering prayers as a cure for HIV.

In a context of medical pluralism, understood as the more or less harmonious co-existence of cultural approaches to sickness and healing [32-34], it is of relevance to investigate the influence of religious beliefs on attitudes to ART. This study gathered FL's perceptions of the alternatives for treating HIV symptoms available in a rural Tanzanian ward. These included traditional, medical and spiritual options.

### Study setting

Kisesa is a semi-rural administrative area of Magu district, in the northwest of Tanzania, comprising a trade centre and six villages some of which are situated along the main road to Kenya, some dispersed in less accessible areas. The predominant ethnic group is Sukuma and most inhabitants are involved in agricultural activities and petty trade. The population of Kisesa was approximately 29.000 inhabitants in 2007 and the approximate annual income per capita is 140$.

In Kisesa there are a total of fourteen primary schools, two secondary schools and seven health facilities of which four are public and three private. There are many traditional healers who are often consulted for health, economic and family-related matters. Traditional religions are reported to be followed by 23% of the population while 74% identify themselves as Christians and 3% as Muslims. Christian denominations present in the area include four mainstream churches (Roman Catholic, Anglican, African Inland Church and the Seventh-day Adventist Church) as well as a wide diversity of revivalists Pentecostal-style congregations, locally known as "Walokole", which have progressively settled in Tanzania since the mid 1980 s and which have become increasingly attractive partly as a result of the networks of healing and care that they offer [15].

Health services in Kisesa are provided by medical practitioners as well as traditional healers and spiritual leaders within a pluralist system in which elements from biomedical and magical/religious traditions are integrated and where different therapeutic modalities and causal explanations for health and illness coexist and interact forming an eclectic approach to disease aetiology. The roll-out of ART is thus taking place in a context in which the new drugs must necessarily compete with a diversity of existing options.

An observational HIV cohort study consisting of a demographic surveillance system (DSS), a community serological survey, and Ante-Natal Clinic surveillance has been operating in Kisesa since 1994. Against the backdrop of this broad study, the TAZAMA project is currently monitoring the roll-out of antiretroviral treatment using both quantitative and qualitative methods. A Voluntary Counselling and Testing (VCT) centre supported by this project has been providing services in the ward since 2005, including a referral system to the ART clinic located at Bugando hospital in Mwanza city [35]. More recently, an ART clinic - also supported by the project - has been established in the local health centre near to the trade centre. Data from the latest serological survey round situates adult HIV prevalence at 7.1% in this area [36].

## Methods

Semi-structured interviews were conducted with 25 FLs recruited from all the villages in the study area. Guidelines for the interviews were developed during a workshop involving all the members of the qualitative research team and the VCT counsellors from Kisesa health centre. The workshop included theoretical and practical sessions on qualitative interviewing with an emphasis on the "funnel approach" in which interviews start with broad questions and are progressively tailored to focus on specific issues spontaneously raised by interviewees. A cyclical research design was used in which the data collected from one interview are analysed immediately and inform the selection of the next faith leaders to be interviewed as well as the development of guidelines for subsequent interviews. This approach allowed us to explore emerging hypotheses in more detail, but required a longer period for the field work activities which lasted a total of 16 months. Informed consent was verbally obtained and tape recorded before each interview took place and 4,000 TSH (~ 3 USD) were provided to participants as a compensation for their time. Ethical approval was obtained from the London School of Hygiene and Tropical Medicine and the Tanzanian Medical Research Consultative Committee.

In close cooperation with the DSS field workers established in each village we identified and invited FLs from the wide diversity of religious denominations present in Kisesa (See Table 1). Sampling took place in two stages. First, we aimed to maximise variation. Subsequently, we purposively selected participants in order to further elaborate emerging hypothesis, some of which were particular to specific denominations. This lead to a high representation of leaders of the Roman Catholic Church and the revivalist churches.

**Table 1:** Study participants by denomination

FAITH DENOMINATIONS	N = 25
***Mainstream churches***	**13**

*Roman Catholic*	7

*Muslim*	1

*Lutheran-Anglican *	2

*Seventh Day Adventists (sabato church)*	1

*African Inland Church*	2

***Revivalist churches ('walokole')***	**12**

*Evangelic Assembly of God of Tanzania (EAGT)*	2

*Pentecostal Assembly of God (PAG)*	1

*Charismatic Evangelical Church of Tanzania (CECT)*	2

*Mitume Church (apostolic church)*	2

*Kanisa la Kristo (the Church of Christ)*	2

*Jesus Miracle Centre *	1

*Nazarene church*	2

While the views of Traditional Healers were not collected in this study our results were analysed in the light of findings from previous research which gathered community's perceptions about THs in the same setting [31].

All the interviews were recorded, transcribed and translated into English. A quality check of a random sample of five documents was conducted in order to maximise the accuracy of the work performed.

**Figure 1:**
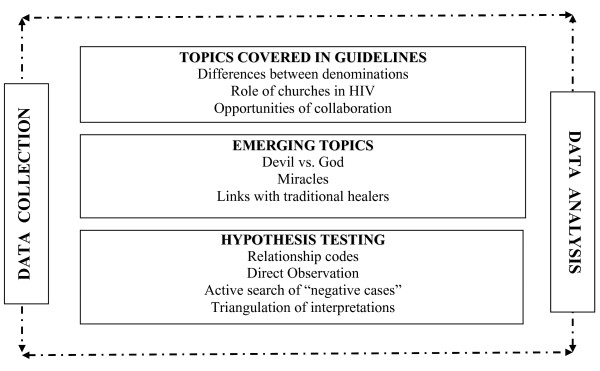
**Data collection and analysis process**.

The analysis process was organised in a continuum of three interlinked stages reflected by the dashed arrows in Figure 1. As the document transcripts became available, the first analyst used an open coding approach that reflected the topics covered in the initial data collection guidelines. Detailed debriefing sessions after each interview allowed us to adapt the guidelines and add new questions immediately on the identification of salient themes. Additional codes were thus created and incorporated to the coding frame to reflect emerging topics. The use of the software package NVIVO-7 helped us to organise the data into themes (see Table 2). Special emphasis was placed on identifying contradictions within respondents' narratives and among members of the same congregation.

**Table 2:** Coding framework

Initial codes	Emerging codes	Relationship codes
**Faith denominations:**	**Devil vs God**	**Links between THs and FLs:**
Differences Interdenominational mobility		Fls perceptions of THs FLs belief in Witchcraft Traditional Healers vs Faith Leaders
**Response of churches to HIV:**	**Miraculous cures:**	**Faith healing as complement of ART**
Support Sin and illness VCT Stigma ART Opportunities for collaboration	Prayers as a cure for HIV Casting spirits away Holy water	**Faith healing as substitute for traditional medicine**

In order to account for one of the more prominent issues that emerged, we coded all the excerpts relating to the links between FLs and THs into a single category which was then further coded to identify sub-themes. Two additional codes were created to analyse in detail the extent to which the different "treatments" available were perceived as alternative or complementary options. To maximise rigour, two additional researchers (fluent in Swahili and/or Sukuma) coded a sample of 12 transcripts of the original data.

In order to contrast the information obtained though individual interviews, several consultations were made with the VCT counsellors working at the health centre in Kisesa and one of the field workers conducted six participatory observation sessions.

A detailed process of discussions aimed at triangulating findings took place among all the researchers and field workers involved in this study. The last stage of analysis consisted of an active search and scrutiny of negative cases and fine-tuning interpretations among team members.

## Results

### Response of churches to HIV: *"Your blood does not match"*

The role of most churches in relation to HIV was dual. On the one hand they advocated against discrimination and provided PLHA with a wide emotional support and a range of practical services. On the other hand, they perceived that in most cases AIDS was a punishment from God for sinful behaviour. Notions of sinful behaviour contributed to the stigmatisation of condoms, which are widely associated with promiscuity.

Many Faith leaders actively promoted VCT uptake among their parishioners. However in the context of pre-marital tests this was often compulsory (*"a law of the church"*) and could impede the marriage celebration if one of the spouses was found to be infected. While a discreet approach was sometimes used to communicate the tests results to the couple (*"your blood does not match"*) confidentiality could also be violated.

*"we sent a letter to the parish priest and the marriage was not officiated" *(FL16-mainstream church)

*"they should submit their results to the priest so that marriage may be sanctioned and then be publicized" *(FL22- mainstream church)

### High inter-denomination mobility: *"Like the movement of safari ants"*

Most FLs agreed that the doctrines of the various denominations present in Kisesa differed very little. There was a high level of inter-denomination mobility and it was mainly guided by pragmatism. The presence of a choir, permissiveness towards alcohol and cigarette consumption, peers' preferences, and the provision of material and emotional support were all factors commonly reported to influence an individual's choice of one denomination over another.

*"we teach the same things... many of them don't come because of personal preference, it is a result of what they hear before they go: 'There is so and so in there'... when one wants to join s/he would consider where many of his/her peers are" *(FL12- revivalist church)

*"the Church is like the movement of safari ants ... some get in and some get out ... the ones I have now are different from those we started with" *(FL11- revivalist church)

### Perceptions of ART: *"the virus now is like a mute person"*

FLs perceptions of ART were characterised by ambivalence. While positive aspects such as *"added years" *were acknowledged, the beneficial effects were sometimes viewed with disappointment, and considered temporary and superficial because the drugs would not offer a definitive cure for HIV. There were also concerns that after a certain period ART would become ineffective and cause a *"sudden death"*. Half of the study participants feared that the availability of ART would contribute to spread the disease further as users regained weight, felt *"normal"*, and could not be physically identified as infected. The following fragments of interviews with FLs from two different mainstream churches reflect some of these concerns which as we have noted elsewhere are frequently raised by villagers in our study setting. [30]

*"now someone takes the medicine and becomes fat, looks fine, s/he'd just perpetuate matters of fornication terribly ... these medicines ... they are risky ... um ... very risky, because someone cannot tell who an infected person is. A person takes these medicines and changes completely ... in the past they'd become so thin ... the virus now is like a mute person" *(FL25-mainstream church)

*"When one sees such a person becoming fat, they think that s/he had been slandered, s/he does not have HIV...and would just go on well practicing sexual intercourse with him/her" *(FL4-mainstream church)

A leader from a revivalist church illustrated the view that the negative attributes of ART drugs could outweigh their benefits:

*"...anti-retroviral medicines ... the medicines are good...on the other hand they are partly good and partly bad because when a person infected with HIV gets the medicines s/he continues to infect others, because people would find him/her healthy and will keep on demanding sexual intercourse from him/her ...they do not observe the instructions given to them by doctors ... that's why they are very harmful... isn't there another method that we could use so that HIV/AIDS is not being transmitted to other people?" *(FL11- revivalist church)

Others would go further and propose measures to ensure villagers would be able to recognize ART users and be *"wary of them"*. Identifying PLHA through external signs like a "special" t-shirt, asking health professionals to publicly disclose infected persons, providing them with *"medicines to reduce the desire for sexual intercourse" *and isolating them in *"specific places" *were among the measures proposed by some of the Faith Leaders interviewed.

*"when one is provided with the medicines ... maybe they should provide T-shirts and force them to wear them maybe for two months in the village ... then we'd know the T-shirt: well ... so this person is like this! ... a whole week for one to wear it ... it'd help somehow and we'd be wary of the person" *(FL12- revivalist church)

In spite of the ambivalent and sometime negative perceptions of consequences of the drugs, these leaders did not report discouraging their use and the use of spiritual treatment appeared to be offered more as a *complement to *than as a *substitute for *ART:

*"I've no problem with people taking the medicines (ART) ... 'saved' people would go to hospital and be provided with these medicines ... but even if one would go to hospital, I'd tell them: 'don't forget to rely on God ... don't forget to pray to God and if possible before you take the medicines, ask God: 'God, may You bless these medicines so they may bring about healing in my body?'... they should know that in spite of taking the medicines death is inevitable... so s/he should take the medicines but s/he should bear in mind that: I'm taking the medicines, but one day I will die ... so when s/he knows that s/he should change his/her life and orient it towards God so that God may forgive his/her sins, then when the day to die comes, s/he'd rest in peace" *(FL10- revivalist church)

### The healing power of faith: *"s/he may test and find HIV is gone"*

FLs from all denominations referred to a cosmology based on a battle between Good and Evil. Misfortune, including disease, was interpreted as a (temporary) triumph of the Devil over God. For some, the Devil could still be defeated through miracles as God would be able to *"heal something that is absolutely impossible for a human being"*. More than 1/3 of study participants reported belief in miraculous cures for HIV including half of the leaders from revivalist churches.

*"...it is true that we have made prayers for people (PLHA) and some of them have healed absolutely right here at the church...we'd been delivering waves of prayers (...) s/he stood in front of the church: 'Hey people, I used to have HIV ' ...the Lord saved him/her... you may pray for him/her and on that minute s/he'd get healed and s/he may go and get tested and find HIV has gone away (...) He told me: 'Do people with HIV get healed?' And I told her: 'They get healed... of course, God cures'. S/he may even tell you: 'Pastor, I'm going to check whether there is HIV in there or God has already cured me?'...when s/he finds He's cured him/her, s/he'd come to tell you: 'Pastor, I've been cured'. God has helped you ... then I thank God" *(FL10 - revivalist church)

*"...this thing about healing is a reality ... we pray for different people with diseases...and they heal...even with this HIV ...people can heal depending on belief. Yes, they heal because when Jesus was over there at the cross, there is no disease he said: 'This disease ... I can't heal it'" *(FL25- mainstream church)

*"...another thing is about trusting in Jesus Christ ... there is such medicine ... it can heal a person who's been infected with HIV ... when Jesus Christ died on the Cross, He came to die for people ... He died for the cure of every type of disease ... when we believe in God, a miracle will take place... so a person would heal someone on a matter that is impossible in human perception (...) there was a person who had been lame for thirty years but when he came to see Jesus Christ and believed in Him he started to walk ... when a person believes in Jesus Christ, God heals him/her ... for God there is nothing that is impossible" *(FL5- revivalist church)

*"HIV is just like other diseases ...when we make prayers, we pray that God provides assistance ... if He wishes that the person gets healed then s/he'll get healed but if the person's fate is to die, however we pray for him/her ... you know, prayers may or may not be answered" *(FL16- mainstream church)

*"if the disease comes from God then the same God has the power to heal a person ... but then I wouldn't say a hundred percent that every person will be healed... even at the time of Jesus Christ not all people were healed ... so even with HIV today, they believe that God heals, but for those He has plans with, not everybody...I've even confirmed some of them ... there is a possibility to be healed from HIV... despite that, I cannot guarantee a hundred percent that everyone coming to the church with HIV will be healed" *(FL12- revivalist church)

*"s/he stood in front of the church: "Hey people, indeed I used to have HIV/AIDS ..." s/he had come here in a critical condition and the Lord saved him/her" *(FL13-revivalist church)

References to miraculous cures described in the Bible were often made and a common view was that prayers would only work for those whose faith was *"strong enough"*. If the healing power of prayers were effective, individuals who had previously tested positive for HIV could even obtain negative results and this would be interpreted as a demonstration that the person had been entirely healed from the disease. However, other FLs did not believe at all in the existence of spiritual cures for HIV and perceived such practices as "cheating" strategies to earn an income, along similar lines to the "tricks" used by some THs:

*"there are some (THs) who are more or less business makers, they do not decline: 'I can't cure this disease' so that s/he may earn something from the patient and get daily needs: 'Come by and I'll perform you a ritual and you will heal'. With religious leaders this kind of small talk might also be present because not every religious leader has a vocation to serve people. Some religions are there to make money" *(FL16- mainstream church)

The effectiveness of prayer as a cure for HIV symptoms was linked to biblical concepts of "sin", "repentance", "mercy", "forgiveness" and "faith":

*"s/he may get the grace of God and live longer and even heal completely ... it depends on how one presents oneself before God:'God. Help me', 'God, have mercy of me' ... of course God cures, healing (of HIV) exists. The first you tell him/her is about sin and you guide him/her through a prayer for forgiveness so s/he may reconcile with God" *(FL10- revivalist church)

As spiritual healing could not be warranted and God had *"only a part to play" *the uncertain outcome of prayer was stressed by respondents and the coexistence of spiritual and biomedical approaches to the disease was commonly found in the respondent's discourses.

*"we believe God is the healer, not for HIV/AIDS only, even for other diseases ... but if a person's belief is not strong enough to believe in God to that extent... let him/her take the medicines (ART) so they may help him/her ..." *(FL13- revivalist church)

*"my advice (to PLHA) is...the first thing to pray to God... s/he may or may not heal ... and then another thing, I'll tell him/her to get a health check-up so s/he may get further treatment" *(FL11- revivalist church)

However, reports from the VCT counsellors providing services in this ward and FLs talking about "others" suggests that the belief in spiritual cures for HIV may lead some ART users to discontinue treatment.

*"you'd find many saying:' Oh, we are saved... then you should not take medicines' ... some people say indeed that when you fall sick you're not supposed to take medicines ... you ought to pray to the Almighty God only... you'd find someone refusing to take medicines: 'let's just pray'" *(FL16- mainstream church)

*"... s/he went to church and was told to abandon all the medicines because they'd pray for him/her but when s/he abandoned the medicines the condition started to deteriorate and up to now his/her health condition hasn't improved" *(interview with VCT counsellors)

The case of Mr. X, the only ART user who died during the 3-year longitudinal follow-up of 17 subjects using ART clearly illustrates how the belief in a spiritual cure for AIDS can contribute to hampering adherence to ART (see Table 3).

**Table 3:** The history of Mr. X: *"I am saved, God has healed me"*

When we first interviewed Mr. X in 2006 he had already initiated ART and was in a good state of physical and emotional health. Mr. X responded closely to what could be categorized as the profile of a "good adherer": he had not experienced side effects, was back to productive activities, had disclosed his HIV status to his wife and was able to economically sustain his family. He had hope for the future and plans to have descendents. He did not feel stigmatized by community members who often approached him asking for advice on HIV-related matters. His increased body weight made him feel optimistic and 'normal' and he reported having changed behaviours in relation to alcohol consumption and extra-marital sex. However, the VCT counsellors operating in Kisesa expressed growing concerns about Mr. X as he was increasingly missing appointments at the ART clinic. Involved in the fishing business, he reported travelling frequently to the islands in Lake Victoria without carrying his medication.

While he used to be a member of a mainstream church he had recently joined a revivalist denomination (*"I went to church to be saved, to the faith healing churches"*). He believed he had been *"healed by God" *during a "laying-on-of-hands" session and attributed his physical recovery to a miracle rather than to the antiretroviral therapy (*"the bishop laid his hand on me"*). By the time health professionals managed to persuade him to return to treatment his state had already deteriorated seriously. He passed away in his thirties, in 2008.

### Links between traditional healers and faith leaders: "*how can we be Christians and remain to be Africans?"*

Traditional healers (TH) were categorised by some FLs as *"agents of evil" *and were often negatively judged for attributing HIV symptoms to witchcraft. However, many of the FLs interviewed acknowledged that villagers often seek out the services of THs, who are credited with the ability to consult the *"divining board of ancestors"*. Through the practice of divination (*"ramli"*) the identity of persons involved in witchcraft practices would be revealed to the healers who would then accuse certain villagers, often closely related to the persons afflicted with disease, as responsible for inflicting it through sorcery. The impact of the THs accusations go beyond weakening extended family support networks: references to killings of individuals accused of sorcery were frequently made throughout the whole period of data collection. One leader from a mainstream church even associated the recent wave of albino murders to the HIV "treatments" prescribed by THs.

*"a pastor and a traditional healer are like water and oil ... there is no association between them ... if you mix them, oil must be afloat, water will remain at the bottom ... as far as the Bible is concerned we are two opposing forces ... s/he must get facilities from the devil even if the society would seem to support him/her ... and as I told you, the work of the devil is to slaughter and destroy" *(FL9- revivalist church)

*"... you take there (to a THs place) an AIDS patient and s/he'd pretend to read a divining board: 'I can see it is your aunt' ... his/her kins would also hear that ... 'she's the one inflicting trouble on our mother or whomever' ... one gets up and chops her... things like that ... or: 'if you want to heal, just go and bring hair of an albino and the disease will be absolutely wiped away'... that's why you'll find killings of these albinos are on the increase...traditional healers ... if they were isolated several miles away ..*." (FL13- revivalist church)

*"...you should not be going to traditional healers ... a person would be told: 'You've been bewitched by so and so'... as a result now we hear of the continuing murdering of people suspected to have bewitched others" *(FL 15- mainstream church)

In spite of this hostility, links between THs and FLs were stronger than initially appeared: a few faith leaders from mainstream churches expressed positive views about the healers' ability to treat certain diseases, the services of FLs and THs were sought by villagers for a similar array of issues, and there was a rumour about a FL from a mainstream church who had learnt *"tricks from the witches"*. We identified frequent contradictory reports that *"church goers" *and even FLs themselves were consulting traditional healers.

*"I was called by God just like how a traditional healer may be called by the Devil... so we have differing perceptions with regards to trusting in God (...) there is a possibility for a pastor to go and see a healer ... it is possible" *(FL12-mainstream church)

*"he is double-minded: he believes in this and he believes in that too...s/he hasn't dedicated his/her whole self to the service of God ... so s/he still believes in these other things too" *(FL11- revivalist church)

In the following excerpt a leader from a mainstream church tried to explain the "disorientation" felt by villagers from a historical perspective illustrating the complex interplay of identity, culture and medicine that often characterises post-colonial societies [32].

*"we have been disoriented from our culture... now looking at this foreign culture that many of us have been embracing and rushing to...it is a challenge that we face: trying to integrate Christianity with our situation as Africans**... **how can we be Christians and remain to be Africans?... that is the very big challenge...our believers would say that traditional healers practice superstition and misguided beliefs but they are the ones who go to see them" *(FL4-mainstream church)

### Casting "bad spirits" away: "*It might be the Devil in attack in form of HIV"*

More than 1/3 of the FLs interviewed explained to us that they believed in witchcraft. Amongst these, some believed that AIDS symptoms could be caused by *"bad spirits" *(*"mapepo"*) inflicted through sorcery. A leader from a mainstream church even described to us the differences between *"real HIV" *and *"mapepo"*-induced AIDS.

*"sorcerers exist and miracles are being performed whereby a person would become sick ... a person who suffers from HIV takes a long time but for someone to whom the disease has been inflicted through sorcery it takes a short period" *(FL16- mainstream church)

However other leaders from the same denomination doubted - or did not believe at all - that witchcraft existed.

Members of both revivalist and mainstream churches described how "the Devil" - via bad spirits - could *"take the form of HIV"*. The "false" virus could imitate the real one so well that the affected person could even obtain a positive HIV result after undergoing a serological test. There was no consensus among study participants, however, over how the source of HIV symptoms could be identified: some would only accept a biomedical diagnosis and others would believe that "mapepo"-induced AIDS could not be identified by "specialists".

*"The thing is about spirits ("mapepo")... Satan could come in the form of HIV and make you wonder: Is this person truly suffering from HIV? When the person gets tested it is HIV, but it may not be HIV, it might be Satan in attack in form of HIV... we'd make prayers to go against it. However, with real HIV no prayers can remove it" *(FL18- revivalist church)

*"you should not come here saying: 'I feel like I've been occupied by genies... they suck my blood and make me become thin'... God cannot heal you because, firstly, you're lying ... you should go for testing so you may be certain" *(FL13- revivalist church)

*"We acknowledge indeed that black forces exist ... you know, these sorcerers and healers... people would look at him/her and think s/he has HIV but it is not HIV. We just pray for this...Government specialist and so forth ...they cannot discover this at all ... that is what we deal with ... someone might be inflicted with diseases ... because of black forces... the devil could inflict certain things...someone would become as thin as if s/he has HIV.... and spirits ... if someone has been occupied with... spirits and goes for testing ... um ... you cannot detect someone has been occupied... that is a different kind of force, you cannot see it ... but if I put my hands on spirits, when someone has been occupied by them, there are signs that will alert me...I'd just approach someone and tell him/her: look into my eyes, I'd be able to see: This person has been occupied by spirits..." *(FL25-mainstream church)

This man also described to us in all detail how he managed to exorcise a woman affected by *"mapepo" *who had been experiencing HIV symptoms for a while:

*"'You demons: today is your day to go away and leave this woman'... she could see something come out ... she had been entered by ... here in the chest, like smoke ... that thing came out ... she's become safe and is in good health ... if you combine my belief and her belief, demons are nothing, diseases are nothing ..." *(FL25- mainstream church)

When HIV symptoms were induced by a "genie" or by "mapepo", prayers could be used as an effective tool to eliminate the disease. In line with the previous quote, other FLs described to us different ways through which spirits could be cast away:

*"for those with strong belief, 'maji ya baraka' (holy water) is of great help...if you're suffering, it helps you...even bad spirits" *(FL19- mainstream church)

*"when we start making prayers to our God, they (demons) run away...the spirit runs away and leaves the person free" *(FL11- revivalist church)

This suggests that some of the "services" provided by THs - like "removing spirits" - are also being offered by some FLs:

"*in most cases they (PLHA) come seeking to get some prayers disguising to have problems, maybe: 'I've been occupied by genies because I experience frequent fevers' ... they started by taking her to THs but...she was lying helplessly in the living room...we prayed for her...she has healed completely" *(FL13- revivalist church)

"*God could tell me: 'if there are sorcerers, do so and so' ... We too make prayers for... a man might be occupied with spirits, well...genies*" (FL10- revivalist church)

Yet these services are being offered in a context of THs deteriorating reputation and a recent ban of their activities across the country, which hampers possibilities for collaboration between traditional and modern providers of health services.

*"the healers should get some training because they've got nothing at all...if you invited them they'd be afraid: 'we are going to be arrested and detained" *(FL11- revivalist church)

## Discussion

We have seen how this rural setting is characterized by high levels of inter-denomination mobility, ambivalent attitudes towards ART and the belief that AIDS symptoms can be caused by witchcraft and be potentially treated through spiritual means.

Summing up our findings, two hypotheses emerged. The first is that spiritual healing is offered more often as a complement than as a substitute for biomedical treatment. Most of the FLs who said that they could cure HIV also mentioned that a positive outcome could not be warranted because healing would depend on the strength of the faith of those involved. As a result of this uncertainty, the use of ART would generally not be discouraged as it could potentially help in cases where prayer had been unsuccessful.

The second hypothesis that emerged is that some of the services offered by FLs are close substitutes for those provided by THs. Traditional healers would attribute the presence of HIV symptoms to "genies" (*"majini"*) or "bad spirits" (*"mapepo"*) and villagers wishing to inflict pain on others would resort to witches (*"wachawi"*) who would perform satanic rituals to attain this (often in the form of an AIDS-like disease). According to Traditional Healers (*"waganga wa jadi"*) divination ("*ramli"*) should then be performed to consult *"the board of ancestors" who *would reveal to the healers who the "witches" are and which rituals should be performed to "remove" the evil spirits and eliminate the disease.

For the FLs, being HIV-positive would generally be seen as *"a punishment from God"*. As the sinful behaviour (promiscuity) originating the *"punishment" *(HIV) could be caused by *"instigations of Satan"*, a "r*econciliation with God" *could be possible. In line with the *"pietistic understanding of sin and forgiveness" *that has been linked to the worship style of revivalist churches [1] the disease could thus be completely cured through prayers *if there was also *true repentance for the sins committed and the persons involved were *"faithful enough"*. This explanation would still be compatible with the biomedical viewpoint which attributes AIDS symptoms to a virus transmitted through risky behaviours and which can affect "anyone" (including those deemed as "innocent" such as the negative spouse of an infected partner or a child infected by its mother). Consequently ART treatment would not be discouraged and by maintaining the uncertainty the church leaders would avoid accusations of raising false hopes [15] while at the same time attracting to their congregations individuals affected by disease. However, several FLs also believed that AIDS symptoms could be caused by *"evil spirits" *which *"imitate" *the HIV virus. Individuals affected by such *"spirits" *could even obtain positive results when undergoing a serological test in spite of not being affected by *"real" *HIV. After undergoing a successful exorcism session the person would be completely healed and test results could even turn out negative.

The fact that some of the FLs interviewed believed that AIDS symptoms could be caused by the devil, or by a real virus and/or a "malevolent spirit" induced by witchcraft exemplifies that no clear-cut boundaries exist between the different systems of beliefs that coexist in this setting and that such boundaries can be easily crossed. Similar processes of "medical integration" have been reported in a diversity of post-colonial societies in which different explanatory models of disease and therapeutic strategies exist alongside each other and borrow concepts from one another, forming complex hybrid systems where elements of conflicting belief systems are integrated [33,34,37] albeit not always without tensions [32,38,39].

**Figure 2:**
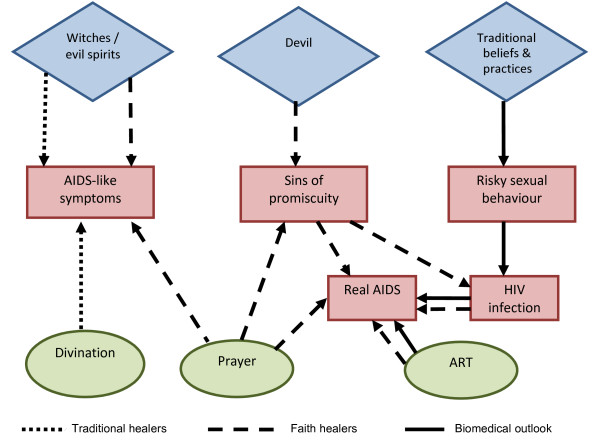
**Aetiology of AIDS symptoms and treatment options available in Kisesa, Tanzania**.

Figure 2 illustrates the different approaches to disease aetiology that coexist in our study setting and which treatment options are being offered by the different providers operating within the pluralistic system. In the figure, diamond shapes represent ultimate causes for the disease, rectangles refer to problems, and circles to different existing alternatives to tackle such problems. As reflected in the figure, the FLs explanations of AIDS symptoms are compatible with both the traditional (malevolent spirits) and biomedical (viral) explanations of the disease. ART treatment was consequently not discouraged and prayers appeared to be offered more as a *complement *than as a *substitute *for antiretroviral drugs.

This hypothesis is consistent with the results of a recent quantitative study also conducted in Tanzania in which no significant associations were found between the belief that a prayer can cure HIV and the hypothetical willingness to initiate ART [1]. However, other studies have shown that actual ART users may discontinue treatment if they believe that they have been spiritually cured through prayers [12,30,31]. Our initial hypothesis thus needs to be further examined in the light of existing knowledge and conflicting evidence from other data sets.

To begin with, we need to bear in mind that our study is based on self-reported data. FLs might not have been willing to explain to researchers from a medical institution that they were discouraging ART particularly in a context in which punitive measures are being instituted against practices related to "occult powers". In addition to the governmental ban on THs activities in Tanzania, the rumour that a FL claiming to cure HIV had been recently arrested in Kenya was reported by one interviewee, and might have contributed to underreporting of the promotion of prayer as the ultimate cure for AIDS. Second, the respondents' support of biomedical options contrasts sharply with recurring concerns over the role of ART users in the continuing spread of the disease. Third, while most FLs might not be supportive of the exclusive use of prayer as treatment for HIV, the claim that spiritual healing can totally remove the disease implies that a potentially superior alternative is being offered and this could *indirectly *lead to ART interruption among users who might *"feel healed"*. As the case of Mr. X shows, the belief in spiritual healing could contribute to hindering treatment adherence and ultimately lead to treatment failure and death. Furthermore, besides offering a definitive cure for HIV, the power of prayer could potentially "erase" the stigma ascribed to the disease as prayers would only be effective for those who are 'faithful enough' and have presumably abandoned 'sin' to turn to God.

As long as the benefits of ART and its complementarity to prayers are not clearly and explicitly stated by FLs, spiritual treatments may be interpreted by some users as a more advantageous alternative. In contexts where ambivalent attitudes towards the new drugs prevail, enhancing FLs understanding of ART's strengths and pitfalls is an essential step to gain their support and engage them as active partners in scale-up programs. Specific actions that they could take might include offering prayers for people to remember to take their medications, for understanding and forgiveness on the part of an uninfected partner, and for the strength of resolve to use condoms to continue to protect loved ones.

Previous successful initiatives engaging faith networks demonstrate that training programs addressed to FLs can yield positive results and that a greater collaboration between the health sector and faith-based organizations (FBOs) is possible [40-43]. In Uganda for example, "treatment literacy" workshops were organized with FLs who subsequently used existing church networks to train other members of their congregations [41]. In Nigeria, religious leaders were sensitised and trained about the importance of ART and the possible negative consequences of non-adherence [40]. In Zambia a series of workshops coordinated by the National Aids Council successfully culminated in the development of guidelines for a greater involvement of traditional and religious leaders in health programming [42]. On the other hand, health providers should be aware of both the positive and negative influence that religious beliefs might have on treatment adherence when providing counselling to ART users.

Moving to our second hypothesis, the belief in witchcraft- induced AIDS-like diseases is not exclusive to Kisesa [19,20] and the performance of rituals to exorcise malevolent spirits *"disguised as a virus" *has been documented in both rural and urban settings across Tanzania [15,22,31]. In the context of a deteriorating reputation of THs [31] and an outright ban on their activities, such rituals appear to be close substitutes for those traditionally offered by diviners. This finding is consistent with previous studies conducted in other SSA countries in which Pentecostal discourses have been categorised as *"an alternative form of witchcraft"*[21] and which link the expansion of revivalist churches to the HIV pandemic [15]. The authors of one of these studies stated that in spite of *"Pentecostal churches' efforts to transcend the power of witchcraft, they in many cases become encompassed by witchcraft discourse, often taking on the appearance of witchcraft itself" *[21]. An ethnography conducted in Uganda, taking as an example a lay organisation of a mainstream church, concluded that *"the Christian anti-witchcraft movements are instrumental in reinstating the occult powers they fight against" *[44]. In line with our findings, the fight against *"the occult" *would paradoxically *"reproduce and strengthen the enemy" *in a context in which *"Christianity has not put an end to witchcraft" *but rather provided *"a new context in which it makes perfect sense" *[44].

## Conclusions

Biomedical and magical-spiritual explanations for HIV symptoms coexist in FLs discourses. While prayers seem to be offered more as a complement than as a substitute for ART, in the absence of strong and explicit support for ART some users may perceive spiritual options as a potentially superior alternative.

The documented tendency to turn to religion after an HIV+ diagnosis and FBOs capabilities to reach rural areas situates FLs in a privileged position to contribute to increasing the local acceptability of the drugs but further efforts should be directed towards enhancing FLs understanding of ART's strengths and pitfalls.

The impact of banning TH's activities on the demand of spiritual services deserves further attention as THs and FLs are consulted for similar reasons and share an economic context of severe deprivation.

This study is constrained by the small number of respondents and reliance on self-reported data. Further research using a quantitative approach is needed to monitor trends of ART uptake and adherence by religious affiliation.

## Competing interests

The authors declare that they have no competing interests.

## Authors' contributions

MR conceived and designed the study, coordinated field work, conducted analysis, and wrote the first draft of the paper. RN conducted most of the interviews and helped to interpret findings. BN facilitated the recruitment of participants and contributed to field work and analysis. JW conducted data analysis and contributed to the interpretation of findings. JB provided technical advice and contributed to drafting the paper. MU, director of the cohort study, helped to coordinate the activities and contributed to drafting the paper. BZ and JT, technical advisors for the broader cohort study, provided overall advice and contributed to drafting. All authors read and approved the final manuscript.

## Pre-publication history

The pre-publication history for this paper can be accessed here:

http://www.biomedcentral.com/1471-2458/10/427/prepub
